# Cytogenomic Profile of Uterine Leiomyoma: In Vivo vs. In Vitro Comparison

**DOI:** 10.3390/biomedicines9121777

**Published:** 2021-11-26

**Authors:** Alla S. Koltsova, Olga A. Efimova, Olga V. Malysheva, Natalia S. Osinovskaya, Thomas Liehr, Ahmed Al-Rikabi, Natalia Yu. Shved, Iskender Yu. Sultanov, Olga G. Chiryaeva, Maria I. Yarmolinskaya, Nikolai I. Polenov, Vladislava V. Kunitsa, Maka I. Kakhiani, Tatyana G. Tral, Gulrukhsor Kh. Tolibova, Olesya N. Bespalova, Igor Yu. Kogan, Andrey S. Glotov, Vladislav S. Baranov, Anna A. Pendina

**Affiliations:** 1 D.O. Ott Research Institute of Obstetrics, Gynecology and Reproductology, 199034 St. Petersburg, Russia; efimova_o82@mail.ru (O.A.E.); omal99@mail.ru (O.V.M.); natosinovskaya@mail.ru (N.S.O.); natashved@mail.ru (N.Y.S.); timbuctu@mail.ru (I.Y.S.); chiryaeva@mail.ru (O.G.C.); m.yarmolinskaya@gmail.com (M.I.Y.); polenovdoc@mail.ru (N.I.P.); vlada.vlada.91@list.ru (V.V.K.); kakhiani74@mail.ru (M.I.K.); ttg.tral@yandex.ru (T.G.T.); gulyatolibova@yandex.ru (G.K.T.); shiggerra@mail.ru (O.N.B.); ikogan@mail.ru (I.Y.K.); anglotov@mail.ru (A.S.G.); baranov@vb2475.spb.edu (V.S.B.); pendina@mail.ru (A.A.P.); 2 Institute of Human Genetics, University Hospital Jena, Friedrich Schiller University, 07747 Jena, Germany; thomas.liehr@med.uni-jena.de (T.L.); ahmedgenetic@hotmail.com (A.A.-R.)

**Keywords:** uterine leiomyoma, abnormal karyotype, chromosomal rearrangements, chromothripsis, *MED12* mutations

## Abstract

We performed a comparative cytogenomic analysis of cultured and uncultured uterine leiomyoma (UL) samples. The experimental approach included karyotyping, aCGH, verification of the detected chromosomal abnormalities by metaphase and interphase FISH, *MED12* mutation analysis and telomere measurement by Q-FISH. An abnormal karyotype was detected in 12 out of 32 cultured UL samples. In five karyotypically abnormal ULs, *MED12* mutations were found. The chromosomal abnormalities in ULs were present mostly by complex rearrangements, including chromothripsis. In both karyotypically normal and abnormal ULs, telomeres were ~40% shorter than in the corresponding myometrium, being possibly prerequisite to chromosomal rearrangements. The uncultured samples of six karyotypically abnormal ULs were checked for the detected chromosomal abnormalities through interphase FISH with individually designed DNA probe sets. All chromosomal abnormalities detected in cultured ULs were found in corresponding uncultured samples. In all tumors, clonal spectra were present by the karyotypically abnormal cell clone/clones which coexisted with karyotypically normal ones, suggesting that chromosomal abnormalities acted as drivers, rather than triggers, of the neoplastic process. In vitro propagation did not cause any changes in the spectrum of the cell clones, but altered their ratio compared to uncultured sample. The alterations were unique for every UL. Compared to its uncultured counterpart, the frequency of chromosomally abnormal cells in the cultured sample was higher in some ULs and lower in others. To summarize, ULs are characterized by both inter- and intratumor genetic heterogeneity. Regardless of its *MED12* status, a tumor may be comprised of clones with and without chromosomal abnormalities. In contrast to the clonal spectrum, which is unique and constant for each UL, the clonal frequency demonstrates up or down shifts under in vitro conditions, most probably determined by the unequal ability of cells with different genetic aberrations to exist outside the body.

## 1. Introduction

Uterine leiomyomas (ULs), also called “fibroids”, are benign smooth-muscle neoplasms of the uterus which are found in 70–80% women of reproductive age [1,2,3]. ULs may cause a significant decrease in life quality due to dysmenorrhea, anaemia and fatigue resulting from heavy menstrual bleeding, as well as non-cyclic pain, abdominal protuberance and other complications [4,5]. In some cases, ULs are associated with reproductive health issues, such as infertility or recurrent miscarriage [6,7,8].

ULs are supposed to be of monoclonal origin [9,10]. However, there remains an ambiguity concerning nature, sequence and actual role of endocrine and paracrine disorders, environmental exposure, as well as genetic and epigenetic changes resulting in the transformation of a myometrium stem cell into a tumor-inducing cell. Hypoxia and early-life exposure to xenoestrogens are considered to be among potentially relevant environmental factors affecting myometrium stem cells through various mechanisms, including epigenetic ones [11,12,13,14,15,16,17]. Genetic changes in ULs vary and typically feature a complex background, including germline (*FH*, *COL4A5-COL4A6*, *PTEN*, *AKT1* mutations, ethnicity-associated genetic variants) and somatic mutations (*MED12*, *HMGA2* mutations and chromosomal abnormalities) [2,18,19,20,21]. The significance of germline mutations for the initiation of tumorigenesis is apparent; by contrast, the input of somatic mutations is still debatable in the sense of whether they are the triggers or the drivers of UL development. Moreover, a tumor may feature single [22,23,24] or multiple genetic changes [25,26], which arguably complicates the investigation of their correlation with the pathogenetic features of these benign neoplasms [27]. However, it should be taken into account that determining the relationship between genetic changes and the impact of different factors, including the in vitro environment, is highly relevant and essential to understanding UL ethiopathogenesis.

For this objective, the present study aimed to investigate genetic abnormalities in ULs in vivo and in vitro using a complex cytogenomic approach: (1) karyotyping of QFH/AcD-stained metaphase chromosomes from cultured UL cells; (2) identification of the breakpoints on metaphase chromosomes with the FISH technique; (3) aCGH analysis of the genetic imbalance in uncultured tumor samples; and (4) a search for chromosomal abnormalities in single cells from both cultured and uncultured samples with the use of the interphase FISH technique. The obtained data provide new insights into how different chromosomal abnormalities affect survival and propagation of UL cells outside the body.

## 2. Materials and Methods

### 2.1. Patients and Samples

**Figure 1:**
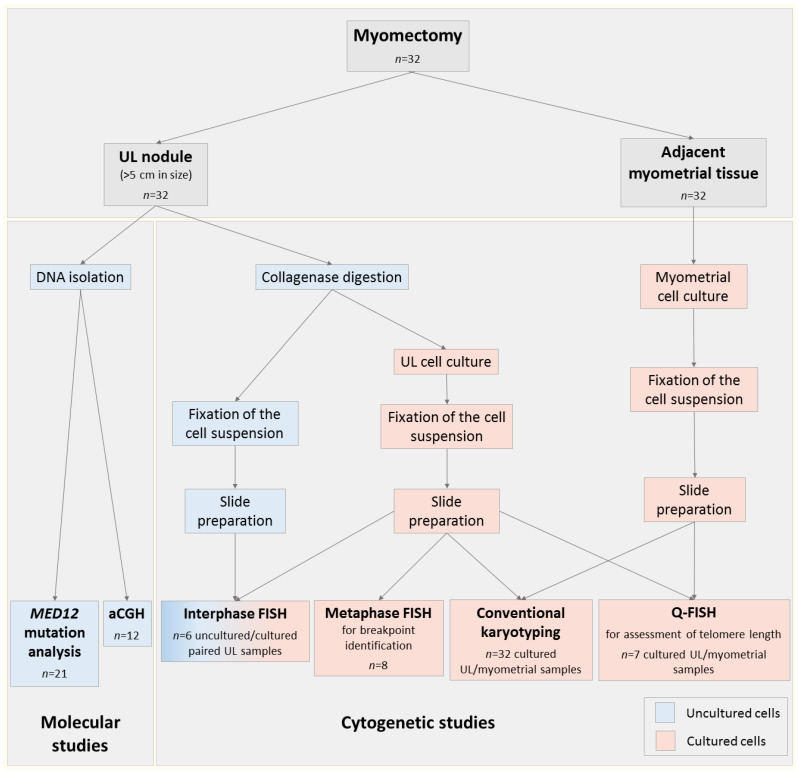
Study design. Immediately after myomectomy, the large uterine leiomyoma (UL) nodule and the adjacent myometrium were sampled. The UL nodule was divided for molecular and cytogenetic studies. Normal myometrium was sampled only for conventional karyotyping and telomere length assessment. For *MED12* mutation analysis and array comparative genomic hybridization (aCGH), DNA was extracted from the fresh tumor tissue. For cytogenetic studies, the cell suspension was obtained by disaggregating fragments of the UL nodule with collagenase. A part of the suspension was fixed, and other part was processed for UL cell culturing. Slide preparations were made from suspensions of cultured and uncultured UL cells. Myometrial tissue was cultured; the cell suspension was fixed, and slide preparations were made. The preparations from cultured UL and myometrial cells underwent both conventional karyotyping (with subsequent metaphase FISH to identify the chromosomal breakpoints) and Q-FISH. The preparations from cultured and uncultured UL cells underwent interphase FISH.

The study enrolled 32 women who underwent myomectomy for large ULs (>5 cm in diameter) at the D.O. Ott Research Institute of Obstetrics, Gynecology and Reproductology (St. Petersburg, Russia) in the period from December 2017 to January 2020. The indications for surgery were pregnancy planning or a symptomatic UL. The patients’ mean age was 38 ± 4.9 years. One UL nodule and one adjacent myometrium sample were obtained from each patient (Figure 1).

### 2.2. Cell Cultures

For UL cell cultures, a fragment of tissue was separated from each UL nodule. The tissue was minced and digested with collagenase according to the previously described protocol [28,29]. Further culturing until passage 1, harvesting and slide preparation were performed as described earlier [30]. 

Cell cultures from the myometrium were obtained without preliminary enzymatic digestion of the tissue samples. In brief, myometrial samples were minced with scissors and placed in flasks with Gibco Gibco^®^ AmnioMAX™ C-100 Complete Medium (Thermo Fisher Scientific, Waltham, MA, USA). The cells attached to the flask surface within the first week. The cells were harvested after 10–14 days at a confluency of 80% and processed for slide preparation according to the abovementioned protocol for UL cell culturing.

### 2.3. Karyotyping of Cell Cultures

Conventional cytogenetic analysis was performed on QFH/AcD-stained metaphases from cultured UL and myometrial cells. From 9 to 100 metaphases (mean 20.3 ± 3 SEM) per UL culture were analyzed at a ≥400-band level.

### 2.4. FISH Studies for Chromosomal Abnormalities

To investigate the structure of aberrant chromosomes, we performed fluorescence in situ hybridisation (FISH) on metaphase preparations from the cultured UL cells using locus-specific, centromeric, or whole chromosome probes. For homemade FISH probes, a previously described protocol was used [31]. For commercial FISH probes, the procedures were performed according to the manufacturer’s recommendations (Abbott Laboratories, Chicago, IL, USA; ZytoVision GmbH, Bremerhaven, Germany; Leica Biosystems, Buffalo Grove, IL, USA) with minor modifications [30]. The FISH probes used to identify the breakpoints on metaphases from karyotypically abnormal ULs are listed in Table S1.

For six karyotypically abnormal ULs, commercial and homemade probe sets were designed for the detected chromosomal abnormalities, and interphase FISH (iFISH) was performed on cultured and uncultured tumor samples. In each cultured and uncultured UL sample, 500–1000 interphase nuclei were analyzed. The FISH signal patterns of each probe set were interpreted by an experienced cytogeneticist, as described in Table 1.

### 2.5. Q-FISH for Assessment of Telomere Length

Telomere lengths were assessed on the metaphase chromosome preparations from UL and myometrial cultures by quantitative FISH (Q-FISH). The *RB1* gene located in the 13q14 region was selected as a reference for telomere length measurements because it is usually non-mutated in ULs and is characterized by low inter-individual variability. For simultaneous detection of telomeric regions and the 13q14 region (the reference region for telomere length measurements in our study), a mixture of telomeric (Telomere PNA FISH/Cy3; DAKO, Glostrup, Denmark) and locus-specific probes (LSI 13 *RB1* 13q14 SpectrumOrange Probe; Abbott Laboratories, USA) was applied. The FISH procedures were performed according to the manufacturer’s protocol for hybridization with telomeric probes, with minor modifications as described in [32].

Post-hybridization fluorescence images of the metaphase plates were acquired using the Leica DM 2500 microscope, the Leica DFC345 FX camera and the Leica Application SuiteV.3.8.0 software (Leica Microsystems GmbH, Wetzlar, Germany) with the following acquisition options: exposure time—2.8 s, gain—×4, gamma—2.00. The FISH signal intensity was evaluated on the digital images using Image J 1.48v software (National Institutes of Health, Bethesda, MD, USA), as described in [33]. To avoid errors associated with different condensation of chromosomes on the metaphase plate, we calculated relative telomere length instead of absolute value. For this, the intensity of the telomeric DNA probe fluorescent signal was measured on the short and the long arm of each chromatid in homologues of chromosome 13 (4 measurements on each homologue). Then, the mean value was calculated and divided by the mean fluorescence intensity of the reference DNA probe on each metaphase plate. In each UL and corresponding myometrial sample, 10–12 metaphases were analysed (Table S2). 

### 2.6. DNA Isolation for Molecular Genetic Studies

DNA was extracted from fresh uncultured UL tissue with the use of the DNeasy Blood and Tissue Kit (Qiagen, Venlo, The Netherlands) according to the manufacturer’s protocol. The DNA concentration was determined with the NanoDrop 2000 spectrophotometer (Thermo Fischer Scientific, Waltham, MA, USA). 

### 2.7. Array Comparative Genomic Hybridisation

The array comparative genomic hybridisation (aCGH) was performed using a G5963 GenetiSure Pre-Screen Microarray 8 × 60K according to the protocol recommended by the manufacturer (Agilent Technologies, Santa Clara, CA, USA). Tumour genomic DNA was co-hybridised with a sex-matched control (Agilent Human Reference Female DNA). aCGH slides were scanned on a ScanRI (PerkinElmer, Waltham, MA, USA), then processed and analysed with Agilent Cytogenomics software v5.2 (Agilent Technologies, Santa Clara, CA, USA).

### 2.8. MED12 Mutation Analysis

*MED12* exon 2 mutations were screened by polymerase chain reaction (PCR) direct sequencing according to a protocol repeatedly used in our laboratory [19,34].

## 3. Results

### 3.1. Karyotype Abnormalities in Cultured ULs Are Represented Primarily by Structural Chromosomal Rearrangements

**Figure 2:**
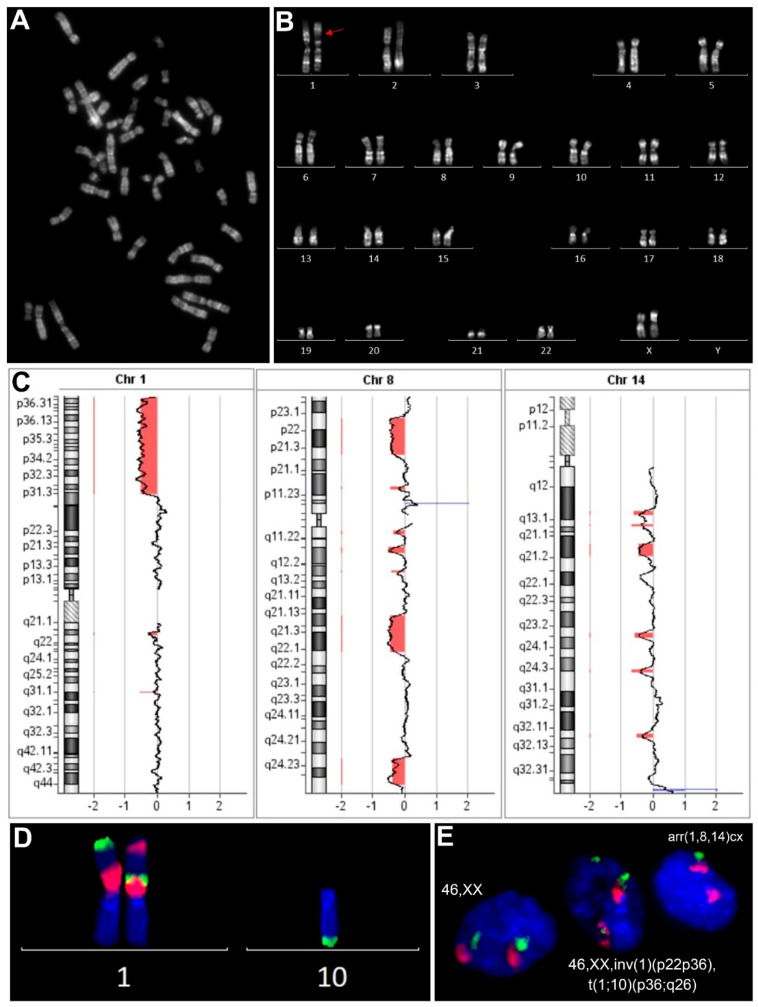
Comprehensive analysis on uterine leiomyoma (UL) karyotype. QFH/AcD-banded metaphase plate (**A**) and corresponding karyogram (**B**) from cultured sample of UL 6. An apparently balanced paracentric inversion of the p22-p36 region in chromosome 1 was detected (red arrow). (**C**) The aCGH of the paired uncultured sample of UL 6 showed changes resembling chromothripsis: multiple deletions alternating with normal segments in chromosomes 1, 8, and 14. (**D**) Metaphase FISH with probe set designed for the detection of both rearrangements (MCB1-1 (1p35.2-1pter, green), MCB1-4 (1p12-1p31.1, red), see Table 1). In cells with a 1p inversion, an additional rearrangement was found: a translocation t(1;10)(p36;q26). (**E**) Representative picture of interphase FISH results: a nucleus with an apparently balanced rearrangement of chromosomes 1 and 10, a nucleus with multiple deletions in chromosomes 1, 8 and 14 and a nucleus without these abnormalities.

In the first stage of the study (Figure 1), we performed a conventional cytogenetic analysis of 32 UL cell cultures (Figure 2A,B), of which 20 (62%) showed a normal (described in [30]), and 12 (38%), an abnormal, karyotype (Table 2). All corresponding cultured myometrial samples had a karyotype of 46,XX. The clinical characteristics of the patients with karyotypically abnormal ULs are given in Table 3.

One UL (1) featured a numerical chromosomal abnormality: trisomy 12. In 11 ULs, karyotype abnormalities were represented as structural chromosomal rearrangements. To specify the structure of the rearranged chromosomes, we used fluorescence in situ hybridisation (FISH) (Figure 2D; Table S1). Three ULs (4, 11, 12) featured a deletion; another one (UL 10), featured a translocation between homologous chromosomes and an insertion. Two more (2, 6) showed apparently balanced reciprocal changes between non-homologous chromosomes, and five ULs (3, 5, 7, 8, 9) had translocations and/or inversions with partial monosomies (Table 2). 

**Figure 3:**
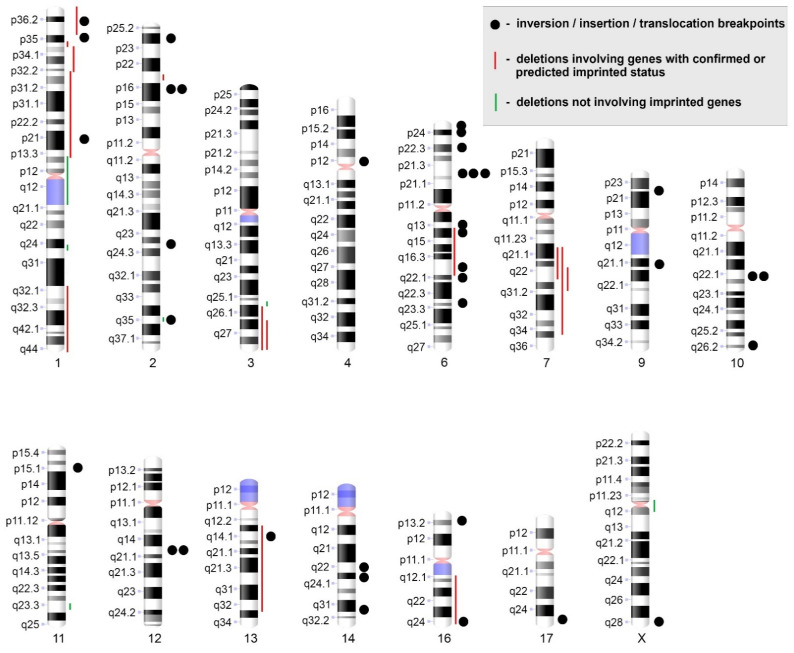
Idiograms of chromosomes involved in the rearrangements in the studied ULs. Apparently balanced rearrangements were present in 25 chromosomal regions, which had been documented earlier, and in 7 novel regions (2p16, 2q24, 4p11, 6q14, 9q21, 10q26, 16q12). A total of 14 out of 20 deleted chromosome regions were comprised of genes with confirmed or predicted imprinted status.

Structural rearrangements involved chromosomes 1, 2, 3, 4, 6, 7, 9, 10, 11, 12, 13, 14, 16, 17 and X (Figure 3). The most frequently observed aberrations were structural abnormalities of the short arm of chromosome 1 (1p) both as independent rearrangements (2/12) (11, 12) and as part of translocations involving other chromosomes (3/12) (6, 7, 8). A total of 3 ULs (2, 9, 10) showed rearrangements of the 6p21 chromosome region. ULs 3, 4, and 5 featured interstitial deletions in the long arm of chromosome 7 (7q); in 1 case, the deletion was accompanied by another rearrangement typical of ULs—a translocation t(12;14)(q15;q24)—and in the other case, by a translocation t(4;?;12;10)(p11;?;q15;q22). Deletions were identified in 1p, 1q, 2p, 2q, 3q, 6q, 7q, 11q, 13q and 16q (Figure 3). Interestingly, we also detected a deletion of one centromere in der(1)t(1;1) in UL 8 and a deletion of the centromeric region of chromosome X in der(9)t(X;9) in UL 9, along with rearrangements of other chromosomes (Table 2). Furthermore, the rearrangement of chromosomes 3 and 13 and both homologues of chromosome 1 found in UL 8 conformed to the criteria of chromothripsis (for more details, see [35]).

Therefore, karyotype abnormalities in cultured UL cells were primarily represented by complex chromosomal rearrangements involving two to seven chromosomes. Breakpoint junctions in reciprocal changes were most frequently observed in chromosome regions 2p16, 6p21, 10q22 and 12q15, while deletions were present primarily in chromosome regions 1p, 3q26~qter and 7q.

### 3.2. Metaphase Chromosome Analysis of Cultured UL Cells Reveals More than One Clone in 50% of the ULs with an Abnormal Karyotype

A total of 6 karyotypically abnormal ULs showed chromosomal abnormalities on all of the metaphase plates, while the remaining 6 showed these abnormalities in 2–78% of examined metaphase plates (Table 2). A chromosomal abnormality was categorised as clonal if identified on two or more metaphase plates.

A total of 6 ULs (4, 6, 7, 10, 11, 12) comprised karyotypically normal metaphase plates (46,XX), along with karyotypically abnormal ones. In these ULs, the number of abnormal clones varied from one to two. Five ULs had only one clone with an abnormal karyotype. In 3 ULs with structural abnormalities in 1p (6, 11, 12), karyotypically normal cells were a predominant, and in UL 7, a minor, subpopulation. UL 4 with a deletion in 7q was characterised by a predominance of the abnormal clone. Finally, UL 10 featured 2 minor clones with unrelated rearrangements.

UL 9 consisted exclusively of karyotypically abnormal cells: an initial clone with a complex rearrangement and several subclones that combined such a rearrangement with other karyotypic abnormalities. As the subclonal proportion and the order in which the rearrangements occurred could not be established, the karyotype of UL 9 was written as composite (Table 2). 

Overall, based on their clonal compositions, conventional karyotyping yielded four groups of karyotypically abnormal ULs: (i) a UL consisting of one karyotypically abnormal cell clone; (ii) a UL consisting of two clones: a karyotypically normal and a karyotypically abnormal one. The abnormal clone can be either predominant or minor; (iii) a UL consisting of three clones: one with a normal karyotype and two differently represented abnormal clones; (iv) a UL consisting of a clone with a basic chromosomal abnormality and subclones combining it with other alterations.

### 3.3. The Telomeric Regions of Metaphase Chromosomes Are Shorter in Both Karyotypically Normal and Abnormal UL Cells than in the Adjacent Myometrium Cells

**Figure 4:**
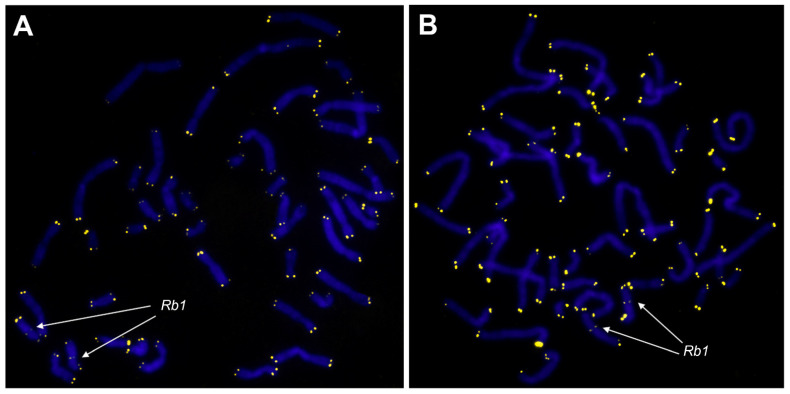
Telomere detection in the metaphase chromosomes from a UL cell (**A**) and a cell of adjacent myometrium (**B**) after in vitro culturing (magnification 10 × 100, var. × 0.63). Telomeres were detected through fluorescence in situ hybridisation (FISH) with telomeric DNA probes (Telomere PNA FISH/Cy3; DAKO, Denmark) and reference locus-specific probes (Vysis LSI 13 RB1 13q14 SpectrumOrange Probe; (Abbott Laboratories, USA). The chromosomes were stained with DAPI.

**Figure 5:**
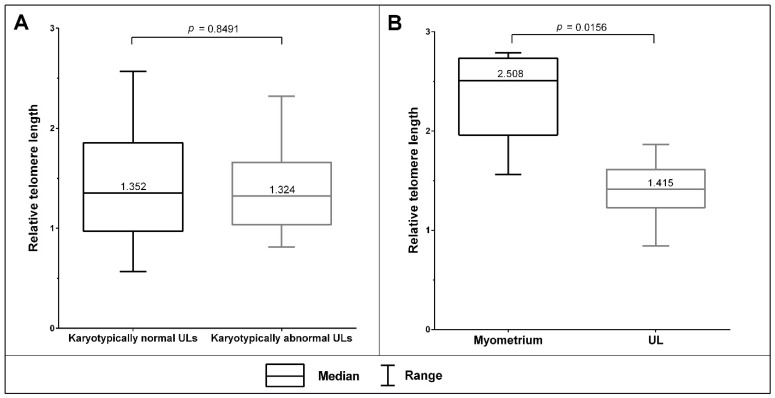
Relative telomere length in cultured ULs and corresponding adjacent myometrium. (**A**) Relative telomere lengths do not differ across the karyotypically normal ULs (*n* = 5, a total of 53 metaphases) and the karyotypically abnormal ULs (*n* = 2, a total of 20 metaphases) (*p* = 0.8491, Mann–Whitney U test). (**B**) Relative telomere lengths are significantly lower in UL samples (*n* = 7) than in the corresponding myometrium (*n* = 7) (*p* = 0.0156, Wilcoxon signed-rank test).

We performed a comparative analysis on five karyotypically normal and two karyotypically abnormal ULs (5, 6) to investigate telomere length in metaphase chromosomes across cultured UL cells and the adjacent normal myometrium (Figure 4). We established an absence of relative telomere length difference between the karyotypically normal ULs (*n* = 5, a total of 53 metaphases) and the abnormal ones (*n* = 2, a total of 20 metaphases) (*p* = 0.8491, Mann–Whitney U test) (Figure 5A). We identified a significant decrease in relative telomere lengths in the ULs (*n* = 7) compared to the corresponding samples of the adjacent myometrium (*n* = 7) (*p* = 0.0156, Wilcoxon signed-rank test) (Figure 5B). Therefore, cultured ULs are characterised by shorter telomeres than the corresponding myometrium. The trend does not depend on the UL’s karyotype.

### 3.4. Most Karyotypically Abnormal ULs Show Discordance between Results of Conventional Karyotyping of Cultured Cells and aCGH Results of Paired Uncultured Samples

Following conventional karyotyping of cultured cells combined with FISH analysis, array comparative genomic hybridisation (aCGH) on corresponding uncultured UL samples from 12 karyotypically abnormal ULs was performed (Figure 2C; Table 2). An absence of imbalance was established for 4 karyotypically abnormal ULs: 2 tumours with apparently balanced chromosomal rearrangements (2, 10), a tumour with non-mosaic trisomy 12 (UL 1) and a UL with a low-clonal (7%) deletion in 1p (UL 12). In the remaining eight karyotypically abnormal ULs, the aCGH revealed an imbalance mostly represented as deletions caused by simple and complex chromosomal rearrangements. In four ULs (3, 4, 5, 8), the deletions detected by aCGH matched the chromosomal imbalances described in metaphase chromosomes from the paired cultured samples (Table 2). The identified imbalances differed from karyotyping results in four ULs (6, 7, 9, 11). In UL 7, aCGH showed an additional chromosomal abnormality: mosaic monosomy 19 (Table 2). In UL 6, aCGH analysis revealed chromothripsis-like changes: multiple deletions alternating with normal segments in chromosomes 1, 8, and 14 (Figure 2C). Meanwhile, conventional karyotyping with subsequent FISH analysis of cultured cells from the same tumour revealed a mosaic (30%) balanced rearrangement involving chromosomes 1 and 10 (Figure 2A,B,D). The aCGH analysis of UL 11 showed deletions in 1q and 12q as well as duplication within the Xp22.31 region, while its conventional karyotyping revealed a deletion in 1p represented in 7.7% of cells (Table 2). In UL 9, the aCGH did not show a centromeric deletion in chromosome X among multiple others (Table 2). Therefore, in 8 cases out of 12 (2, 3, 5, 6, 7, 8, 9, 10), the conventional karyotyping identified apparently balanced chromosomal abnormalities that could not have been identified by aCGH. In one case (UL 1), conventional karyotyping showed a numerical chromosomal abnormality that the aCGH analysis did not detect. Furthermore, in three cases (6, 7, 11), aCGH on uncultured UL samples revealed an imbalance that had not been detected by the conventional karyotyping of the paired cultured samples.

Overall, combining conventional karyotyping techniques, FISH and aCGH in the analysis of cultured and uncultured UL cell samples allows for a more accurate description of a UL’s cytogenomic profile.

### 3.5. Chromosomal Abnormalities Detected by Conventional Karyotyping and aCGH Are Present in the Interphase Cells of Uncultured ULs

**Figure 6:**
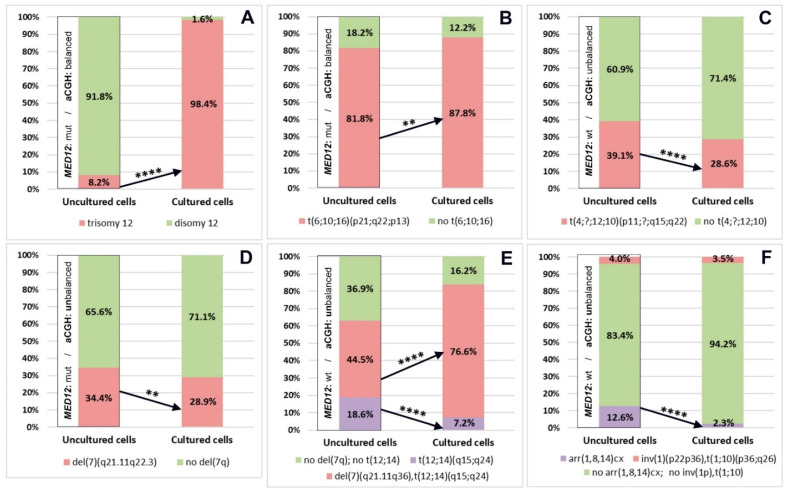
A shift of the initial clone ratio in karyotypically abnormal ULs caused by cell culturing. Abnormal clones with trisomy 12 and a translocation t(6;10;16)(p21;q22;p13) in ULs 1 (**A**) and 2 (**B**), respectively, became significantly more expressed after in vitro propagation. The sizes of abnormal clones with a translocation t(4;?;12;10)(p11;?;q15;q22) and a del(7)(q21.11q22.3) in ULs 3 (**C**) and 4 (**D**), respectively, decreased significantly under culture conditions. In UL 5 (**E**) with two related abnormal clones, the frequency of cells with a translocation t(12;14)(q15;q24) was significantly lower in the cultured sample. In contrast, the frequency of cells with a translocation t(12;14) and a deletion in 7q was significantly higher. In UL 6 (**F**) with 2 unrelated abnormal clones, the frequency of cells with multiple deletions in chromosomes 1, 8, 14 was significantly lower in the cultured sample. In contrast, the frequency of cells with an apparently balanced rearrangement of chromosomes 1 and 10 remained on par with the uncultured sample. Chi-square with Yates’ correction; ** *p* < 0.01, **** *p* < 0.0001.

Subsequently, we applied interphase FISH (iFISH) to the uncultured UL samples to verify the chromosomal abnormalities detected by conventional karyotyping and aCGH (Figure 2E). We designed DNA probe sets capable of validating chromosomal abnormalities in interphase nuclei for 6 out of 12 karyotypically abnormal ULs (Table 1). The iFISH revealed both chromosomally normal and chromosomally abnormal cells in all six analysed ULs (Figure 6). ULs 1, 2, 3 and 4 had two types of cells: those with and those without chromosomal abnormalities. For UL 3 with karyotype 46,XX,del(7)(q22.1q31.2),t(4;?;12;10)(p11;?;q15;q22)[15], a FISH probe set capable of validating a translocation t(4;?;12;10) but not a deletion del(7)(q22.1q31.2) was applied. In UL 5 with karyotype 46,XX,del(7)(q21.1q35),t(12;14)(q15;q23)[15], iFISH revealed 3 types of cells: (i) only translocation t(12;14); (ii) a translocation t(12;14) and a deletion in 7q and (iii) no such chromosomal abnormalities. This suggested that the deletion in 7q was a secondary event in cells with the translocation t(12;14). UL 6 with discordant results between karyotyping and aCGH also had three types of cells: (i) a translocation t(1;10) combined with an inversion in 1p; (ii) multiple deletions in chromosomes 1, 8 and 14 and (iii) no such chromosomal abnormalities.

Therefore, in all six cases, the chromosomal abnormalities identified by conventional karyotyping of cultured UL cells were confirmed to be present in the corresponding uncultured UL samples. Notably, all of the ULs showed a combination of chromosomally normal and abnormal cells. For particular complex chromosomal rearrangements (CCRs), a combination of conventional and molecular cytogenetic methods can help establish the sequence in which the chromosomal abnormalities emerged.

### 3.6. The Initial Proportions of Normal and Abnormal Clones Established in Native ULs Alter after In Vitro Propagation

A comparative analysis of the number of cells with chromosomal abnormalities in the paired cultured and uncultured samples of six ULs was done next. In UL 1 with trisomy 12, the frequency of abnormal cells was significantly higher in the cultured sample than in the uncultured one (*p* < 0.0001, chi-squared test with Yates’ correction). Remarkably, the clone with trisomy 12, which accounted for 8.2% of cells in the uncultured UL sample, reached a frequency of 98.4% after in vitro propagation (Figure 6A). In UL 2, the frequency of cells with a translocation t(6;10;16) was significantly higher in the cultured sample than in the uncultured one (*p* = 0.002, chi-squared test with Yates’ correction). Both paired samples showed a predominance of cells with chromosomal abnormalities (Figure 6B). In UL 3, the frequency of cells with a translocation t(4;?;12;10) was significantly lower in the cultured sample than in the uncultured one (*p* < 0.0001, chi-squared test with Yates’ correction). Both paired samples contained under 40% of abnormal cells (Figure 6C). In UL 4, the frequency of cells with a deletion del(7)(q21.11q22.3) was significantly lower in the cultured sample than in the uncultured one (*p* = 0.002, chi-squared test with Yates’ correction). Both paired samples comprised under 35% of abnormal cells (Figure 6D). In both cultured and uncultured samples of UL 5, which had 2 abnormal cell clones, the clone with a translocation t(12;14) accounted for under 20%, whereas the subclone with a translocation t(12;14) and a deletion in 7q reached a frequency of over 45%. In the cultured sample, the frequency of cells with a translocation t(12;14) was significantly lower than in the uncultured one (*p* < 0.0001, chi-squared test with Yates’ correction), whereas the frequency of cells combining karyotype abnormalities—a translocation and a deletion—was significantly higher (*p* < 0.0001, chi-squared test with Yates’ correction) (Figure 6E). In UL 6, which contained 2 abnormal clones—(i) with an apparently balanced rearrangement involving chromosomes 1 and 10 and (ii) with multiple deletions in chromosomes 1, 8 and 14—the frequency of cells with multiple deletions was significantly lower in the cultured sample than in the uncultured one (<0.0001, chi-squared test with Yates’ correction). However, the frequency of cells with the apparently balanced rearrangement involving chromosomes 1 and 10 remained unchanged. In either sample, cells with chromosomal abnormalities did not account for over 13% (Figure 6F).

We investigated the relationship between the shifts in the normal and the abnormal clone proportion in vitro and the balanced/unbalanced condition of chromosomal abnormalities in five ULs (1, 2, 4, 5, 6). In the cases of both the balanced karyotype abormalities (t(6;10;16)(p21;q22;p13) in UL 2; t(12;14)(q15;q24) in UL 5; inv(1)(p22p36),t(1;10)(p36;q26) in UL 6) and the imbalanced ones (trisomy 12 in UL 1; del(7)(q21.11q22.3) in UL 4; del(7)(q21.1q35),t(12;14)(q15;q23) in UL 5; arr(1,8,14)cth in UL 6), the proportion of clones shifted either towards a higher or a lower frequency of the abnormal clone (Figure 6).

Therefore, a change in the initial clonal proportion in the ULs with chromosomal abnormalities after in vitro propagation was evident. However, the spectrum of karyotypically different clones and the changes in their proportions were unique to each UL and did not depend on the balanced/unbalanced condition of the chromosomal abnormality.

### 3.7. MED12 Gene Mutations Do Not Rule Out the Presence of Karyotype Abnormalities in ULs 

The *MED12* status was analysed in ULs with a normal (*n* = 9) and an abnormal (*n* = 12) karyotype. In 5 out of 12 karyotypically abnormal ULs, we detected heterozygous missense mutations in exon 2, while the remaining 7 tumours showed no *MED12* mutations. A total of 4 ULs (1, 2, 4, 12) had a c.131G>A substitution in codon 44 (*p*. G44D) and UL 10 had a c.107T>G substitution in codon 36 (*p*. L36R) (Table 2). In karyotypically normal ULs, *MED12* mutations were detected in four out of nine tumours [30]. The *MED12* mutation frequency did not differ between ULs with a normal and an abnormal karyotype (*p* = 1.000, Fisher’s exact test). However, we observed a tendency towards *MED12* mutations in the karyotypically abnormal ULs with no imbalance revealed by the aCGH. Therefore, *MED12* mutations were detected with an equal frequency in karyotypically normal and karyotypically abnormal ULs.

### 3.8. A UL’s MED12 Status Is Not Associated with Differences in the Proportion of Cytogenetically Normal and Abnormal Clones between Cultured and Uncultured Samples 

Out of 6 karyotypically abnormal ULs subjected to a comparative analysis of the abnormal clone proportion across paired cultured and uncultured samples, 3 tumours (1, 2, 4) had missense mutations in codon 44 of *MED12* exon 2, while the remainders were *MED12*-negative (3, 5, 6). In two *MED12*-positive ULs (1, 2), the frequency of chromosomally abnormal cells was significantly higher in the cultured sample than in the uncultured one (Figure 6A,B). By contrast, another *MED12*-positive tumour, UL 4, had a significantly lower frequency of chromosomally abnormal cells in the cultured sample than in the uncultured one (Figure 6D). The karyotypically abnormal *MED12*-negative ULs also showed different shifts in the clonal proportion after in vitro propagation. Thus, two *MED12*-negative ULs (3, 6) contained a significantly lower frequency of cells with chromosomal abnormalities in the cultured sample than in the uncultured one (Figure 6C,F). By contrast, UL 5 featured a significantly higher frequency of chromosomally abnormal cells in the cultured sample than in the uncultured one (Figure 6E). Therefore, no association between *MED12* status of a UL and differences in the proportion of karyotypically different cell clones after in vitro propagation could be identified.

## 4. Discussion

Investigating UL karyotypes has been made possible by the advancement of tumour cell culture techniques, which detect chromosomal abnormalities in up to 50% of ULs [36,37,38,39]. The present study revealed an abnormal karyotype in 38% of cultured ULs primarily represented by structural and, less frequently, numerical chromosomal abnormalities. Breakpoint junctions occurred in regions 1p, 6p21, 7q, 10q22, 12q15 and 14q24, which had been frequently referenced in the literature, in regions which had only a few documented cases [36,37,39,40,41,42] and in regions which had not been previously documented (2p16, 2q24, 4p11, 6q14, 9q21, 10q26, and 16q12). Unbalanced structural rearrangements were accompanied by deletions of varying length with an almost complete absence of duplications, which is typical of genetic alterations occurring in ULs [43,44]. The frequent occurrence of deletions in 1p, 3q and 7q suggests that they may harbour tumour suppressor genes and the loss of one allele may facilitate UL growth [2,45,46,47,48,49]. Intriguingly, most of the detected deletions (14 out of 20, Figure 3) involved the location regions of imprinted genes or genes with a predicted imprinting effect [50,51]. 

Gene imprinting disorders caused by either the loss of a functional allele or an epigenetic mutation, particularly aberrant DNA methylation, occur in various types of malignant neoplasia [52]. However, it remains unclear why the disrupted imprinting of the same genes may be associated either with malignant or benign tumorigenesis, in particular in ULs. Presumably, the main contributing factors are the type and location of cells in which the disorder occurs. We also cannot rule out the presence of functional allele protection mechanisms in ULs, which cause deletions to affect mostly non-functional (imprinted) alleles and do not result in a catastrophic loss of function.

The deletions affected not only chromosome arms but, in some cases (ULs 8 and 9), one of the two centromeric regions in rearranged chromosomes. The loss of a centromeric region in a dicentric chromosome decreases the risk of chromosome loss during cell division and represents a chromosome stabilisation mechanism [53]. The formation of dicentrics and other chromosomal rearrangements per se may be caused by a cell crisis that results from intensive divisions during tumor progression and leads to telomere shortening [54,55,56]. In the present study, having compared telomere lengths of chromosomes from cultured ULs to those from the adjacent myometrium, we have indeed found evidence that both karyotypically normal and abnormal ULs are characterised by shorter (on an average of 40%) telomeres than the adjacent myometrium. Therefore, we suggest that telomere shortening is typical of ULs and most likely precedes the occurrence of chromosomal aberrations in UL cells. The rejuvenation of telomere length does not appear to be characteristic of ULs either through telomerase activity [57,58] due to the lack of an active telomerase complex [59,60] or through the ALT mechanism, which is typical of malignant tumours [61]. Therefore, telomere shortening in UL cells is most likely a factor contributing to chromosomal aberrations. 

Karyotype alterations are specific to ULs, but their detection and study are possible only in metaphase chromosomes of cycling cells obtained through culturing [62,63]. Consequently, a legitimate question is whether chromosomal abnormalities detected through karyotyping emerge in vivo or in vitro. Using different cytogenomic approaches, we demonstrated that all chromosomal abnormalities detected by karyotyping of cultured cells were present in paired uncultured samples of examined ULs, while all corresponding cultured myometrial samples were karyotypically normal. A total of 2 groups used aCGH [64] and iFISH [64,65] and demonstrated that rearrangements of chromosomes 7 and 12 detected in cultured ULs were present in native ULs. Taking together, these results provide evidence of chromosomal changes in ULs occurring directly in the tumour and not during culturing.

Interestingly, aberrations may occur either sequentially, as in UL 5, where the translocation (t(12;14)) was a primary event followed by the deletion (del7q), or simultaneously, as in UL 6, where inv(1),t(1;10) and complex rearrangements of chromosomes 1, 8 and 14 resembling chromothripsis emerged independently from each other. A number of studies demonstrated features of UL karyotype evolution [37,40,45,66,67,68,69]. However, benign UL is more cytogenetically stable compared to its malignant counterpart—leiomyosarcoma [41,70,71,72,73,74,75].

Another important result of the study is proof of the in vitro viability of cells with chromosomal aberrations. A novel finding is that in vitro conditions do not alter the clonal spectrum but cause shifts in the initial clonal proportion. Hayashi et al., 1996, demonstrated a growth in the proportion of cells with structural rearrangements of chromosome 12 in cultured compared to uncultured UL samples [65]. Another study reported a tendency to disappear during culturing for cells with 7q deletion [66]. Holzmann et al., 2014 demonstrated the inability of UL cells with chromothripsis to survive in vitro [22], which is partially in line with our observations. The changes established by the present study varied in direction and were unique to each examined UL. Compared to an uncultured sample, its cultured counterpart featured either a higher (ULs 1, 2, 5) or a lower frequency of karyotypically abnormal cells (ULs 3, 4, 5, 6). The obtained results have not allowed us to reveal a correlation between the clonal proportion changes in vitro and either a balanced/unbalanced condition of chromosomal abnormality or *MED12* mutation status. Interestingly, driver mutations and chromosomal abnormalities can be mutually exclusive in ULs [24,76], or co-exist in one nodule [23,25,26]. The interrelation of cytogenetic and molecular genetic abnormalities should be examined in single UL cells, which is a challenging methodological task but is undoubtedly crucial to understanding tumorigenesis processes.

Importantly, the present study establishes that in native ULs, cells with chromosomal abnormalities coexist with cells without such abnormalities. Similar observations were made by other authors [64,65,77,78,79,80]. Therefore, chromosomal aberrations most likely occur after the UL’s formation—possibly during its intensive growth. It should be pointed out that, according to the clinical and medical history data of several patients involved in the study, rapid UL growth was detected a few years after the tumour was identified (Table 3). The fact that tumours in individual patients may exist for a long time without considerably growing could indicate that at some point in time, chromosomally normal and abnormal clones reach equilibrium in their synergistic cooperation, most probably through paracrine mechanisms.

The explosive growth of a UL [81,82,83] accompanied by overexpression of multiple genes [84,85] may favour chromosomal rearrangements. In such cases, actively transcribed genes that are located on different chromosomes may become topologically closer to each other in the transcription factories, which, in turn, may result in faulty repair of multiple DNA breaks and the emergence of structural rearrangements [86,87,88,89,90]. Such a mechanism may underlie translocations involving the 12q15 region—one of the most frequent chromosome aberrations in ULs. Breakpoints at 12q15 are located within the *HMGA2* locus and are associated with *HMGA2* gene overexpression [91,92]. However, *HMGA2* overexpression can be found in karyotypically normal ULs [93], suggesting that *HMGA2* overexpression, on the one hand, can cause rearrangements within the 12q15 region in some ULs. On the other hand, *HMGA2* overexpression itself can be possibly provided by different mechanisms: epigenetic aberrations, mutations, and chromosomal rearrangements. Therefore, an interesting direction for the study is a search for causal links between gene overexpression and its mutations/rearrangements in UL cells.

To summarise, genetic changes in ULs are variant and characterised by a pronounced intra- and inter-tumour heterogeneity. Regardless of its *MED12* mutation status, a single tumour may combine clones with different chromosomal abnormalities and without such abnormalities. Chromosomal abnormalities are most likely to be the drivers rather than the triggers of tumorigenesis. A novel and important finding is that in vitro conditions do not cause changes in the clonal spectrum but alter the initial clonal proportion. These up or down shifts under in vitro conditions are possibly determined by the unequal ability of cells with specific genetic aberrations to exist outside the body. Understanding the relationship between the genetic changes and the ability of cells with such changes to exist in different conditions could be extremely beneficial for exploring the fundamentals of UL tumorigenesis. Moreover, detailed knowledge on the behaviour of karyotypically abnormal tumour cells could contribute to developing new treatment approaches, including personalised ones. 

## Figures and Tables

**Table 1:** FISH probe sets designed for the identification of chromosomal abnormalities in interphase nuclei.

Case	Probe Mix	Cytogenetic Localisation	Manufacturer	Analysis (Detection of Chromosomal Abnormality)
1	CEP 12 (D12Z3)	12p11.1–q11 Alpha Satellite DNA	Abbott Laboratories	There may be three signals of the CEP 12 (D12Z3) probe.
2	MCB6-2 MCB10-5a MCB16-1a	6p24.1–p21.33 10q22.1–q25.2 16p13.3–p11.1	Homemade	The signals of MCB10–5a and MCB16–1a probes may become juxtaposed on one chromosome because of the t(6;10;16)(p21;q22;q13).
3	MCB10-4a MCB12-5	10q11.23–10q23.1 12q15–12q23.3	Homemade	The signals may become juxtaposed on one chromosome because of the t(4;?;12;10).
4	LSI ELN/LSI D7S486, D7S522	7q11.23, 7q31	Abbott Laboratories	The signals of LSI ELN and LSI D7S486, D7S522 probes may become juxtaposed on one chromosome because of the del(7)(q21.11q23).
5	MCB7-5 MCB12-5 MCB14-1a	7q21.11–7q31.2 12q15–12q23.3 14q13.3–14q23.3	Homemade	The signals of MCB12–5 and MCB14–1a probes may become juxtaposed on one chromosome because of the t(12;14)(q15;q24); one signal of MCB7–5 probe may be absent because of the del(7)(q21.11q33).
6	MCB1-1 MCB1-4	1p35.2–1pter 1p12–1p31.1	Homemade	(1) One additional signal of both MCB1–1 and MCB1–4 probes may be present, and they may become juxtaposed because of the inv(1)(p22p36) and the t(1;10)(p36;q26). (2) One signal of the MCB1–1 probe may be absent because of the del(1)(p33).

**Table 2:** The results of array comparative genomic hybridisation (aCGH), *MED12* mutation analysis and interphase FISH for uterine leiomyomas with abnormal karyotype. CTS, cultured tumor sample; UTS, uncultured tumor sample.

Case	Conventional Karyotyping of CTS (with Subsequent FISH on Metaphase Chromosomes)	aCGH of UTS	*MED12* Status of UTS	Interphase FISH on CTS and UTS
1	47,XX, + 12[18]	arr(X,1-22) × 2	wt/c.131G > A, pG44D	UTS: nuc ish(D12Z3×3)[82/1000] CTS: nuc ish(D12Z3×3)[985/1001]
2	46,XX,t(6;10;16)(p21;q22;p13)[13]	arr(X,1-22) × 2	wt/c.131G > A, pG44D	UTS: nuc ish(MCB6-2,MCB10-5a,MCB16-1a)×2(MCB10-5a con MCB16-1a×1)[454/555] CTS: nuc ish(MCB6-2,MCB10-5a,MCB16-1a)×2(MCB10-5a con MCB16-1a×1)[448/510]
3	46,XX,del(7)(q22.1q31.2),t(4;?;12;10)(p11;?;q15;q22)[15]	arr[GRCh37] 7q22.1q31.2(98726412_115199215)×1[0,7]	wt/wt	UTS: nuc ish(MCB10-4a,MCB12-5)×2(MCB10-4a con MCB12-5×1)[391/1000] CTS: nuc ish(MCB10-4a,MCB12-5)×2(MCB10-4a con MCB12-5×1)[286/1000]
4	46,XX,del(7)(q21.11q22.3)[7]/46,XX[2]	arr[GRCh37] 7q21.11q22.3(83605684_105796277)×1	wt/c.131G > A, pG44D	UTS: nuc ish(ELN,D7S486)×2(ELN con D7S486×1)[350/1016] CTS: nuc ish(ELN,D7S486)×2(ELN con D7S486×1)[297/1028]
5	46,XX,del(7)(q21.1q35),t(12;14)(q15;q23)[15]	arr[GRCh37] 7q21.11q35(78201649_146170074)×1[0,6]	wt/wt	UTS: nuc ish(MCB7-5×1,MCB12-5×2,MCB14-1a×2)(MCB12-5 con MCB14-1a×1)[445/1000],(MCB7-5,MCB12-5,MCB14-1a)×2(MCB12-5 con MCB14-1a×1)[186/1000] CTS: nuc ish(MCB7-5×1,MCB12-5×2,MCB14-1a×2)(MCB12-5 con MCB14-1a×1)[766/1000],(MCB7-5,MCB12-5,MCB14-1a)×2(MCB12-5 con MCB14-1a×1)[72/1000]
6	46,XX,inv(1)(p36p21),t(1;10)(p36;q26)[9]/46,XX[21]	arr[GRCh37] (1,8,14)cx[0,6]	wt/wt	UTS: nuc ish(MCB1-1×1,MCB1-4×2)[126/1000],(MCB1-1,MCB1-4)×3(MCB1-1 con MCB1-4×1)[40/1000] CTS: nuc ish(MCB1-1,MCB1-4)×3(MCB1-1 con MCB1-4×1)[35/1000],(MCB1-1×1,MCB1-4×2)[23/1000]
7	46,XX,del(1)(p34p32),del(3)(q26),del(16)(q12q24),t(1;17)(p35;q25),t(2;9)(p16;q21)[25]/46,XX[14]	arr[GRCh37] 1p34.3p32.3(36643269_52009701)×1[0,8],3q13.31q21.1(116742856_122583187)×1[0,8],3q24q26.33(147591180_180696172)×1[0,8],16q12.1q22.1(48779768_69195217)×1[0,8],16q23.2q24.1(79678725_85191053)×1[0,8],(19)×1[0,4]	wt/wt	no
8	45,XX,der(1)t(1;1),der(3)t(1;3),der(13)t(1;3;13),-1[12]	arr[GRCh37] 1p35.1p34.3(34079411_36223052)×1[0,7],1p13.2p12(113262062_120527194)×1[0,7],1q24.3q25.1(172004633_174625860)×1[0,7],1q32.1q44(205440703_249208145)×1[0,7],3q25.32q25.33(158746175_160073609)×1[0,7],3q26.31q29(173336557_197771082)×1[0,7],13q13.1q33.1(32365197_101748020)×1[0,7]	wt/wt	no
9	45,X,-X,der(2)t(2;11)(2p16→2q24::11p15.1→11pter),der(6)(6pter→6p24::6p21→6q14.1::6q22.1→6qter),der(9)t(X;9)(Xpter→Xp11.1::Xq11.1→Xq26::9p22→9qter),der(11)t(2;11)(2qter→2q35::2p24→2p16::2q35→2q24::11p15.1→11qter),der(14)t(6;14)(14pter→14q22::6p24→6p?23::6p?22.1→6p?21),der(16)t(16;6;14)(16p→16qter::6p?22.3→6p?22.1::14q22→14q31.3::6p?23→6p?22::14q31.3→14qter)[cp25]	arr[GRCh37] 2p25.1(8446980_8682420)×1[0,7],2p24.3(12646763_13128408)×1[0,7],2p24.3(14202109_14930579)×1[0,6],2p21(42190927_43424758)×1[0,7],2p21(45127208_45383273)×1[0,7],2q35(218424429_219280813)×1[0,7],2q36.1(224312511_224706849)×1[0,7],6q14.1(81386902_81640806)×1 [0,7],6q14.1(82451363_82888958)×1[0,7],6q14.1q22.1(83806540-114940922)×1[0,7],6q24.1(139618109_140460727)×1[0,7],14q24.1(69062128_69249764)×1[0,7,16p11.2p11.1(32637849_34721199)×3,16q24.2(87466743_87533166)×1	wt/wt	no
10	46,XX,t(6;6)(p21;p25)[2]/46,XX,ins(13;6)(q14;q23q13)[8]/46,XX[90]	arr(X,1-22)×2	wt/c.107T > G, pL36R	no
11	46,XX,del(1)(p32p13)[2]/46,XX[24]	arr[GRCh37] 1q41q43(220523143_241093232)×1[0,5],12q22(93893845_94451632)×1[0,8],Xp22.31(6456036_8152935)×3	wt/wt	no
12	46,XX,del(1)(p36)[2]/46,XX[28]	arr(X,1-22)×2	wt/c.131G > A, pG44D	no

**Table 3:** Patient and uterine leiomyoma (UL) characteristics.

Case	Patient’s Age, Years	Hormonal Treatment before Myomectomy	Menstrual Cycle Phase at the Time of Myomectomy	Solitary (S) or Multiple (M) ULs	Diameter of Analyzed UL Nodule, cm	Localisation of Analyzed UL Nodule (FIGO)	Time Elapsed between UL Diagnosis and Myomectomy	Rapid UL Growth within One Year before Myomectomy	Histological Examination
1	32	No	Proliferative	M	7	5	6 years	yes	Leiomyoma with necrosis
2	45	Ulipristal acetate	Hormonal treatment	S	8	4	5 years	yes	Leiomyoma with oedema and hyalinosis
3	40	No	Proliferative	S	9	6	5 years	no	Leiomyoma with edema
4	43	Buserelin acetate	Hormonal treatment	M	8	4	13 years	yes	Leiomyoma with fibrohyalinosis
5	29	No	Proliferative	S	8	5	1 year	yes	Leiomyoma
6	42	No	Secretory	S	5	4	7 months	no	Leiomyoma with hyalinosis
7	31	No	Secretory	M	9	6	2 months	no	Leiomyoma
8	44	Triptorelin acetate	Hormonal treatment	S	7	4	5 years	no	Leiomyoma with sclerosis and hyalinosis
9	41	No	Proliferative	M	10	6	6 years	yes	Leiomyoma
10	32	No	Proliferative	S	5	4	1 year	no	Leiomyoma
11	36	Cyproterone + Ethinylestradiol	Hormonal treatment	S	5	4	5 years	no	Leiomyoma
12	37	No	Secretory	M	5	2	2 years	no	Leiomyoma

## Data Availability

Not applicable.

## Supplementary Materials

The following are available online at https://www.mdpi.com/article/10.3390/biomedicines9121777/s1, Table S1: FISH probes used to identify chromosomes and chromosome regions involved in the rearrangements, Table S2: Relative telomere length in uterine leiomyoma and paired myometrium cultured samples from patients with normal and abnormal tumour karyotypes.
