# Supplementary material for: Cytogenomic Profile of Uterine Leiomyoma: In Vivo vs. In Vitro Comparison
Source: Biomedicines. 2021 Nov 26;9(12):1777. doi: 10.3390/biomedicines9121777 (PMC8698342; doi:10.3390/biomedicines9121777)
Supplement: Supplementary file 1 [file biomedicines-09-01777-s001.zip › Koltsova et al_Table S1.pdf]

**Supplementary Table S1.** Whole chromosome (WCP or XCP), centromere- (CEP or SE), band- (MCB), arm- (pcp) or locus-specific (LSI) FISH probes used to identify chromosomes and chromosomal regions involved in the rearrangements (except probes listed in Materials and Methods, Table 1).

| Case | Probe | Chromosome localisation | Manufacturer |
|---|---|---|---|
| 2 | WCP6 | Chr. 6 | Applied Spectral Imaging |
| | WCP10 | Chr. 10 | Applied Spectral Imaging |
| | XCP16 | Chr. 16 | Metasystems |
| 3 | WCP4 | Chr. 4 | Homemade |
| | WCP9 | Chr. 9 | Applied Spectral Imaging |
| | WCP10 | Chr. 10 | Homemade |
| | WCP12 | Chr. 12 | Homemade |
| | WCP22 | Chr. 22 | Homemade |
| | WCPX | Chr. X | Applied Spectral Imaging |
| | MCB7 | Chr. 7 | Homemade |
| | MCB10 | Chr. 10 | Homemade |
| 4 | LSI ELN / LSI D7S486, D7S522 | 7q11.23, 7q31.1-31.3 | Abbott Molecular |
| | LSI: RP11-90N9 | 7q21.11 | Homemade |
| | LSI: RP11-313N23 | 7q21.2 | Homemade |
| 5 | WCP12 | Chr. 12 | Homemade |
| | WCP14 | Chr. 14 | Homemade |
| | LSI ELN / LSI D7S486, D7S522 | 7q11.23, 7q31 | Abbott Molecular |
| | MCB7 | Chr. 7 | Homemade |
| 6 | WCP1 | Chr. 1 | Applied Spectral Imaging |
| | LSI 1p36 | 1pTEL, 1p36, 1q25 | Abbott Molecular |
| | MCB1 | Chr. 1 | Homemade |
| | MCB10 | Chr. 10 | Homemade |
| | pcp1p | 1p | Homemade |
| | pcp10q | 10q | Homemade |
| 7 | WCP1 | Chr. 1 | Applied Spectral Imaging |
| | WCP2 | Chr. 2 | Kreatech |
| | WCP3 | Chr. 3 | Applied Spectral Imaging |
| | WCP9 | Chr. 9 | Applied Spectral Imaging |
| | WCP16 | Chr. 16 | Applied Spectral Imaging |
| | WCP17 | Chr. 17 | Applied Spectral Imaging |
| | LSI 1p36 | 1pTEL, 1p36, 1q25 | Abbott Molecular |
| | LSI N-MYC | 2p24 | Abbott Molecular |
| | CEP17 (D17Z1) | 17p11.1-q11.1 Alpha Satellite DNA | Abbott Molecular |
| | LSI: TelVision 17q | 17qTEL (D17S928) | Abbott Molecular |
| | LSI: TelVision 3p and 3q | 3pTEL (D3S4559), 3qTEL (D3S4560) | Abbott Molecular |
| | LSI: TelVision 16p and 16q | 16pTEL, 16qTEL | Abbott Molecular |
| 8 | WCP 1 | Chr. 1 | Applied Spectral Imaging |
| | WCP 3 | Chr. 3 | Applied Spectral Imaging |
| | WCP 13 | Chr. 13 | Abbott Molecular |
| | CEP 1 (D1Z5) | 1p11.1-q11.1 Alpha Satellite DNA | Abbott Molecular |

| | | | |
|---|---|---|---|
| | CEP 3 (D3Z1) | 3p11.1-q11.1 Alpha Satellite DNA | Abbott Molecular |
| | SE13/21 | 13p11.1-q11.1, 21p11.1-q11.1 | Kreatech |
| | LSI 1p36 | 1pTEL, 1p36, 1q25 | Abbott Molecular |
| 9 | WCP2 | Chr. 2 | Kreatech |
| | WCP6 | Chr. 6 | Applied Spectral Imaging |
| | WCP9 | Chr. 9 | Applied Spectral Imaging |
| | WCP11 | Chr. 11 | Applied Spectral Imaging |
| | WCP14 | Chr. 14 | Applied Spectral Imaging |
| | XCP16 | Chr. 16 | Metasystems |
| | WCPX | Chr. X | Applied Spectral Imaging |
| | pcp2q | 2q | Homemade |
| | pcp6p | 6p | Homemade |
| | pcp6q | 6q | Homemade |
| | pcp11p | 11p | Homemade |
| | MCB2 | Chr. 2 | Homemade |
| | MCB6 | Chr. 6 | Homemade |
| | MCB11 | Chr. 11 | Homemade |
| | MCB14 | Chr. 14 | Homemade |
| | MCBX | Chr. X | Homemade |
| | LSI/CEP/pcp: Subcentromere-oriented mix 9 | Chr. 9 | Homemade |
| | LSI: ZytoLight® SPEC EML4 Dual Color Break Apart Probe | 2p21 | ZytoVision GmbH |
| | LSI: ZytoLight® SPEC FOXO1/PAX3 Dual Color Single Fusion Probe | 2q36.1, 13q14.11 | ZytoVision GmbH |
| | LSI: RP11-130P22 | 2p21 | Homemade |
| | LSI: RP11-134D9 | 2p16.3 | Homemade |
| | LSI: RP11-355B11 | 2p15 | Homemade |
| | LSI: RP11-576F1 | 2p21 | Homemade |
| | LSI: RP11-438B23 | 9p21 | Homemade |
| | LSI: RP11-503K16 | 9p22.1 | Homemade |
| | LSI: RP11-513M16 | 9p22.1 | Homemade |
| | LSI: RP11-6K5 | 11p15 | Homemade |
| | LSI: RP11-140J4 | 11p15 | Homemade |
