# Supplementary material for: Cytogenomic Profile of Uterine Leiomyoma: In Vivo vs. In Vitro Comparison
Source: Biomedicines. 2021 Nov 26;9(12):1777. doi: 10.3390/biomedicines9121777 (PMC8698342; doi:10.3390/biomedicines9121777)
Supplement: Supplementary file 1 [file biomedicines-09-01777-s001.zip › Koltsova et al_Table S2.pdf]

Table S2. Relative telomere length in uterine leiomyoma (UL) and paired myometrium (Myo) cultured samples from patients with normal (13, 14, 15, 16, 17) and abnormal (5, 6) tumour karyotype.

Tumour karyotype	Abnormal				Normal									
Case	5		6		13		14		15		16		17	
Sample	UL	Myo	UL	Myo	UL	Myo	UL	Myo	UL	Myo	UL	Myo	UL	Myo
Relative telomere length of both chromosomes 13 on metaphase plate	1.507	3.332	1.305	2.706	1.536	2.131	1.688	1.844	1.453	2.029	2.155	2.823	0.954	1.267
	1.307	1.848	0.968	2.254	2.177	1.673	2.207	2.741	1.521	1.165	0.979	2.317	0.729	1.278
	1.978	3.617	1.936	3.613	1.964	2.392	1.190	2.128	1.469	3.002	0.963	2.794	0.753	1.814
	1.840	2.717	1.000	2.547	2.569	2.263	2.007	2.627	1.268	2.801	0.865	2.056	0.675	1.760
	1.340	2.717	1.046	2.737	1.903	1.590	1.237	1.935	2.218	1.401	0.991	2.545	1.246	1.310
	1.461	3.057	1.139	1.791	1.695	2.399	1.342	1.843	1.384	1.916	0.680	2.946	0.566	1.863
	1.033	1.509	1.447	2.222	1.345	3.764	1.856	3.249	1.684	1.781	1.295	2.604	0.721	1.380
	0.813	2.162	1.026	3.670	2.024	3.082	1.796	1.202	0.850	1.950	0.945	1.844	1.137	1.386
	1.711	3.651	2.322	3.765	1.559	3.061	1.148	1.862	1.874	1.645	1.340	2.835	0.705	1.905
	1.156	3.289	1.504	2.018	1.883	2.811	1.609	2.833	1.352	1.901	2.181	2.314	0.944	1.634
						2.571	1.431				1.114			
						4.710	1.854							
Relative telomere length of the sample (mean value)	1.415	2.790	1.369	2.732	1.866	2.704	1.614	2.226	1.507	1.959	1.228	2.508	0.843	1.560
